# Models of Abnormal Scarring

**DOI:** 10.1155/2013/423147

**Published:** 2013-09-03

**Authors:** Bommie F. Seo, Jun Yong Lee, Sung-No Jung

**Affiliations:** Department of Plastic and Reconstructive Surgery, Uijeongbu St. Mary's Hospital, College of Medicine, Catholic University of Korea, 65-1 Kumoh-Dong, Uijeongbu 480-135, Republic of Korea

## Abstract

Keloids and hypertrophic scars are thick, raised dermal scars, caused by derailing of the normal scarring process. Extensive research on such abnormal scarring has been done; however, these being refractory disorders specific to humans, it has been difficult to establish a universal animal model. A wide variety of animal models have been used. These include the athymic mouse, rats, rabbits, and pigs. Although these models have provided valuable insight into abnormal scarring, there is currently still no ideal model. This paper reviews the models that have been developed.

## 1. Introduction

Keloids are defined as pathologic scars that grow beyond the confines of the original injury [1]. They occur in areas of cutaneous injury, and they are benign, dermal fibroproliferative tumors, with no malignant potential [2, 3]. They are characterized by an excessive deposition of extracellular matrix components, namely, collage, fibronectin, elastin, proteoglycans, and growth factors. There is a higher incidence among African-Americans, Asian-Americans, Latin-Americans, and other darker pigmented ethnicities. Reports of familial cases and parallelism in identical twins imply a genetic contribution to the pathophysiology [4]. Keloids cause cosmetic deformities but are usually asymptomatic. However, some may grow large enough to cause functional limitations, especially when located along the joint. 

The term “keloid” is derived from the Greek word *chele*, which means crab's claw, a comparison to the horizontal growth of the tissue into the normal skin [2]. This characteristic, among others, differentiates them from hypertrophic scars. Hypertrophic scars are fibrous tissue outgrowth with excessive scarring, which are confined to the original wound margins [5]. These scars usually develop within a couple of months after initial wound development, grow rapidly for several months, and then gradually regress over the next few years. Keloids may develop far after initial injury, persist for extensive periods of time, and usually do not regress. Keloids also have increased fibroblast density and proliferation rates, larger, thicker, more wavy collagen fibers, and an increased ratio of type I to type III collagens, unlike hypertrophic scars [6–8]. Hypertrophic scars contain more type III collagen, fibers oriented more parallel to the epidermal surface, with nodules consisting of myofibroblasts. They slowly process through the normal healing cycle of inflammation, proliferation, and maturation, while the keloid scar does not [9]. 

Keloids and hypertrophic scars are one of the most infuriating clinical problems in wound healing. There is still no single, unified hypothesis that explains the pathogenesis of keloid formation. This fact is underscored by the multiple treatment options for keloids including excision, intralesional steroids, adjuvant radiation therapy, laser, silicone, pressure therapy, and combination therapies [10–15]. Some have advocated that intralesional triamcinolone, surgical excision, and radiation actually promote recurrence [2, 10, 16]. 

The suggested hypotheses for the derailing of normal scar formation in keloids are alteration in growth factors and extracellular matrix components via epithelial keratinocyte-mesenchymal fibroblast interactions or hypoxia-induced angiogenetic responses, alteration in collagen turnover, alteration in the keloid fibroblast response to various growth factors, mechanical tension-induced fibroblast proliferation and collagen synthesis, genetic immune dysfunction, and immunological reaction to sebum [17–22]. A multifactorial inheritance model may be the underlying cause of such alterations [4].

Animal models provide valuable translational vehicles for human treatment modalities. However, as these aberrant scars are specific to humans, the development of an animal model for hypertrophic scarring or keloids has been extremely difficult. A major difference between laboratory animals and humans is the presence of the panniculus carnosus in animals, a fibromuscular layer enabling the skin to slide over underlying fascia. This facilitates rapid contraction and faster healing of burn wounds.

The lack of a universal animal model for such scarring has been an obstacle in developing a successful therapeutic strategy. Many groups have attempted to generate animal models, and this collective process has helped to elucidate most of what we now know of this perplexing entity. In this paper, we describe the wide variety of animal models that have been developed and used in the history of scar research.

## 2. Experimental Models of Abnormal Scarring

Keloid or hypertrophic scars have been extensively studied through two types of basic research approaches, either through animal models or through tissue or cell cultures. In earlier research, combined studies on both hypertrophic and keloid scars were common because differentiation between these two entities had not been established.

### 2.1. Animal Models

 There are two main approaches in the development of the animal model. The earlier approach is to induce an innate keloid-like scar in animals. However, such scars were only maintained for a very limited time period and usually developed characteristics of hypertrophic scars. The other approach is to transplant human keloid or hypertrophic scar tissue into animals. Recent studies have integrated tissue engineering with animal models, transplanting cultured human keloid tissue into animals for longer survival.

#### 2.1.1. Induction of Abnormal Scars

Different methods of wounding have been inflicted upon various animals to induce hypertrophic or keloid scars. In 2001, Sullivan et al. performed a review of the literature to compare the adequacy of laboratory animals as a model for human wound healing. Studies on wound dressings, topical antimicrobials, and growth factors using humans, pigs, rabbits, guinea pigs, rats, and mice were examined. The authors found 78% agreement between humans and pigs, 53% agreement between humans and small mammals, and 57% agreement between humans and *in vitro* studies [23]. 

*(1) Pigs. *The first animal model was suggested in 1972 and 1976 by Silverstein who inflicted repeated deep dermal injuries on the backs of twelve red Duroc pigs to induce successful formation of a hypertrophic scar in all the animals. Pigs are tight-skinned animals that have similar skin architecture with humans. They also sustain sunburn and rely on fat for insulation as do humans, unlike other animals that require fur. Both humans and pigs have thick epidermis with similar turnover times. Both have elastic fibers in their epidermis and contain Langerhans cells. Collagen structure is also similar [24]. 

The first model by Silverstein vanished in the literature after being reported. In 2003, Zhu et al. and, in 2004, Gallant-Behm et al. made full-thickness wounds with Padgett dermatomes on the back of female red Duroc pigs, and showed that this animal model presented hypertrophic scarring up to 5 months after the incisions [25]. 

Zhu et al., in their 2003 study, reported that red Duroc pigs had skin cones and developed hypertrophic scars histologically similar to humans. Subsequent studies by the group reported that immunohistochemical patterns of decorin, versican, and insulin-like growth factor-1 (IGF-1) were also analogous to hypertrophic scars in humans and that the number of nerve fibers in the scar was similar [26, 27]. Biochemical studies demonstrated that comparable levels of vascular endothelial growth factor and nitric oxide could be found in human and porcine scar tissue, as were the corresponding numbers of mastocytes, collagen nodules, and myofibroblasts [28, 29].

Gallant-Behm et al., in their 2004 study, compared scar formation in the female red Duroc pigs with that in Yorkshire pigs and juvenile castrated male red Duroc pigs. Gross and histologic results were indistinguishable between the male and female red Duroc pigs. However, expression of types I and III collagen, heat shock protein 47, bone morphogenic protein 1, diverse proteoglycans, and osteopontin differed in pattern, with the red Duroc pig exhibiting a unique biphasic pattern, undocumented previously [30]. Subsequent studies by the same group using electric dermatome wounds on the backs of red Duroc pigs revealed characteristics in expression of cytokines, transcription factors, growth factors, and receptors similar to human scars [31]. Stewart et al., also from this group, reported in 2006 that the kinetics of blood flow in the red Duroc model were comparable with the previously observed laser Doppler imaging of human skin wounds and hypertrophic scars [32]. In 2007, Gallant-Behm's group studied first generation offspring between the red Duroc and Yorkshire pigs. They had intermediate scar behavior, supporting growing evidence that wound phenotypes were genetically programmed [33].

 While porcine skin resembles human skin in many aspects and may develop scars similar in some characteristics to human hypertrophic scars, there are still many limitations to the porcine model. Skin structure is not identical. Pig epidermis has only three layers as opposed to five in humans, with a thick and compact stratum corneum. The distribution of apocrine and eccrine sweat glands are different, as is the architecture of hair follicles. Pigs are also large, costly to obtain, and difficult to maintain [34]. 

*(2) Mice.* In 1983, Ehrlich reported on the hyperplastic wound healing process noted in tight-skin mice. Tight-skin mice (TSM), a mutant mouse strain, have a skin covering tightly adhered to their bodies and were used by the authors to overcome the major contributor to loose skinned animal wound healing: wound contraction. Sharp, full-thickness excision was done to make square defects on the dorsum of tight-skinned mice; wounds were left undressed and were excised at weekly intervals between 1 and 9 weeks following wounding. Abundant swirls of collagen fibers and hypertrophy of connective tissue and vessels histologically similar to human hypertrophic scars were noted [35]. However, scars failed to maintain these characteristics for prolonged periods of time. 

*(3) Rabbits.* In 1997, a group of surgeons at Northwestern University who had noticed that surgical scars in rabbit ears remained elevated months after wounding standardized a rabbit ear model for biochemical and molecular scarring studies. Forty excisional wounds each 6 mm in diameter were created down to bare cartilage on the ventral surface of young adult female New Zealander white rabbit ears. In the acute model, these wounds were treated with either intralesional triamcinolone or saline at day 16 and histologically analyzed at day 22. In the chronic form, larger excisions were made and accumulation of new collagen and cartilage was observed for over 9 months [36]. This model was used and validated in a variety of studies evaluating the effect of age on scars, efficacy of therapeutic agents, and molecular mechanisms [37–39]. 

*(4) Guinea Pigs.* In 2002, Aksoy et al. developed a guinea pig hypertrophic scar model, using albino male guinea pigs and coal tar. They focused on the costly maintenance necessary for immunosuppressed mice or pigs and suggested a cheaper, easier method. Circular skin defects with diameters ranging 1.7~2.0 cm were made on each side of the dorsal thorax, followed by circular defects of the panniculus carnosus with a larger diameter. Any latissimus dorsi muscle remaining between the skin and thoracic wall was removed. The defect on the right was left untouched, and the defect on the right received applications of coal tar every 48 hours, beginning four days after the wounds were made. Scars with erythema and elevated edges developed in 10 out of 12 animals. However, morphological correlation was found in only six of these, and increased glucose-6-phosphate dehydrogenase (G6PD) enzyme activity was only detected in four. G6PD activity is increased in human proliferative scars [36]. Although less costly and easier to conduct than the athymic mouse or pig model, the toxic, carcinogenic effects of coal tar must also be investigated. The longevity of the guinea pigs was not mentioned. 

The most critical limitation of the aforementioned models is that they are all animal models that develop characteristics of hypertrophic scars. Keloids are, to date, virtually impossible to induce in animal wounds. 

#### 2.1.2. Heterologous Transplantation of Human Keloid or Hypertrophic Scar Tissue

 Xenografts of human scar tissue into different wounds of different species have been described. To avoid rejection, either immunodeficient animals such as athymic mice and rats or an immune privileged site such as the cheek pouch of a hamster has been used. 

*(1) Athymic Mouse.* Most studies use variations of the original nude mouse model, incorporating deepithelialized, diced human abnormal scar tissue in the subcutaneous pockets on the back or thorax of nude, athymic mice, with minor alterations. This model is relatively easy to perform; nude athymic mice are relatively easy to maintain, and the implants are easily accessible and visible. 

The athymic mouse was first described in 1966 by Flanagan, and it is still extensively used for transplantation and graft studies thanks to its impaired T-cell function [40].

Shetlar et al. first described implantation of human keloid tissue into subcutaneous pockets of athymic mice in 1985 [41]. In 1987, Robb et al. grafted human cadaveric partial thickness skin to full-thickness skin defects on the backs of athymic nude mice, suturing them to the defect margins. Three weeks after grafting, they created burns which resulted in scars of increased collagen. They also grafted human hypertrophic scars from burn patients to full-thickness skin defects on athymic nude mice, and they found that these grafts were able to revascularize in samples up to 3 mm thick and maintained histologic and gross characteristics for up to 6 months, when the animals were sacrificed [42].

In 1989, Kischer et al. reported on implants of human hypertrophic scars and keloids into the subcutaneous pockets above the panniculus muscle on backs of athymic nude mice and observed growth of the implanted tissue for up to 246 days. Microvascular anastamosis between the grafted scar tissue and host vessels was noted within the first several days. Size reduction was noted, with a slope of −0.436 for hypertrophic scars and −0.736 for keloids. This means that in about 67 days the keloid implants have half of their initial volume. Histological analysis confirmed retention of character. Occlusion of microvessels was consistently seen in transmission electron microscopy [43, 44].

In 1991, Waki et al. used the same model to report on the effects of pharmacologic agents. Deepithelialized human keloids were implanted bilaterally in the subcutaneous pouch of the thorax in athymic mice. They noticed an initial growth spurt until the fourth week after implantation, then regression, a pattern that differed from previous studies. Rejection or collagen degradation, outgrowth of vascular supply, or loss of collagen synthesis gene regulator feedback was suggested as the mechanism [45].

In 2004, an *in vivo* model with genetically modified skin-humanized mice was proposed. Previous studies using genetically modified human skin grafted onto mice had focused on time point analysis of graft behavior and take [46–49]. Cultured bioengineered skin equivalent with labeled keratinocytes was transplanted on the backs of nude mice, and a small, circular full-thickness wound was made 9 to 12 weeks after grafting. The study shows that this model recapitulates the features of native human wound healing, using epithelial and stromal markers [50]. Yang et al. also grafted full-thickness human skin onto full-thickness defects measuring 2.0 × 1.5 cm on the backs of nude mice and inflicted burn injuries after complete graft take to induce scarring. Hypertrophic scars similar to human hypertrophic scars were noted [51]. 

In 2013, Ishiko et al. sutured explanted keloid tissue to the dorsum of the mice to evaluate the effects of chondroitinase injection on keloid tissue. They describe significant reduction in keloid scar tissue volume. The mechanism proposed was the reorganization of the extracellular matrix with regenerated elastic fibers [52]. This method is also used for studies on hypertrophic scars [53, 54]. 

The main limitations of the nude mouse model are small animal size, therefore small sample size, along with difficulty of maintenance on an acceptable, isolated pedicle, and limited longevity. 

*(2) Rat.* An athymic hairless mutation in a colony of outbred hooded rats was first observed in 1953 at the Rowett Research Institute in Aberdeen. Homozygous mutants were recovered in 1975, and in 1977, a breeding colony of congenitally athymic, nude rats were developed [55]. 

Polo et al., noting the limitations of the nude mouse in scar studies, developed a nude rat sandwich flap keloid scar model. They implanted homogeneous sections of human keloid tissue beneath the epigastric island flap of a nude rat and, then, after a 3-week maturation period, elevated the epigastric flap along with the implanted scar tissue. A catheter was inserted into the flap pedicle for future injection purposes. Wrapping the elevated flap around the scar tissue to form a sandwich island flap, the authors passed the flap through a subcutaneous tunnel to the dorsum of the rat. The flap was sutured to two incisions made prior to tunneling [56–58]. This model ensured that the scar tissue was separated from surrounding tissue and supplied, through a single pedicle, the superficial inferior epigastric pedicle. These efforts were made in an attempt to prevent the previously noted absorption of keloid scar tissue, and they resulted in maintenance of the transplanted tissue up to 18 months. Transpositioning to the dorsum allows accurate measurement, isolation of the pedicle allows manipulation of vascular supply, and catheter placement enables intralesional injections minimizing systemic spread [59]. 

*(3) Hamster.* In 2005, Hochman et al. implanted keloid scars from the breast of an adult female patient into both cheek pouches of 18 male Syrian golden hamsters (*Mesocricetus auratus*). This small mammal has a normal immune system, but it is endowed with an immune privileged site, the subepithelium of its jugal pouches. The jugal pouches are diverticular structures inside the mouth used for storing and transporting food. The subepithelium lacks lymphatic structures except in its proximal region, and thus this area has been used in various animal models for grafts and neoplasms [60–62]. Because the epithelium is transparent, the keloid specimens were visible from the mouth. The grafts were analyzed 5, 12, 21, 42, 84, and 168 days after implantation. Histological evaluation revealed increased vascularity, deposition of inflammatory infiltrate and collagen analogous with human hypertrophic scarring. They also noted an increase of melanocytes in the groups sacrificed after 42 days. Unfortunately, epithelium integrity was not completely maintained in the groups after 42 days, and the authors suggest that this model may be effective for about 21 days [63]. 

Transplantation of human keloid or hypertrophic scar xenografts allows us to perform studies on tissue that possesses similar histological structures and biochemical features with *in vivo* scars. However, because the physiological microenvironment differs, such similarities begin to diminish with the passage of time. The human tissue is completely isolated from its *in vivo* environment. Viability is limited, so long term effects of treatment modalities are difficult to assess. The transplanted tissue is usually obtained months or years after injury or onset, so heterogenous material is usually transplanted, and studies on preventive measures are impossible. The cost and energy it takes to maintain and handle immunodeficient rodents is also something to consider [64]. 

### 2.2. Tissue or Cell Cultures

The difficulty in developing a universal animal model is reflected in the abundant research upon cell or tissue culture models of abnormal scars. 

The cell culture technique is to harvest dermal derived cells such as fibroblasts, endothelial cells, and keratinocytes from human keloid scars and culture them two-dimensionally on a plastic substrate or culture dish [65–67]. Early models used serum containing culture media. Because serum contains growth factors, it was used to sustain cell growth, but it confounded the experimental results. In 1997, Koch et al. developed a serum-free keloid fibroblast culture that did not compromise cell growth, enabling evaluation of various growth factors and wound modulators [67]. The major limitation of such cultures is that the cells grown in monolayer cannot replicate the complex cell-to-cell or cell-to-matrix interactions found in intact tissue. 

Tissue cultures have been employed to better study these pathophysiological mechanisms. To better mimic the *in vivo* microenvironmental condition, keloid tissue derived cells have been loaded onto three-dimensional (3D) synthetic scaffolds or grown in a 3D format [68, 69]. Organotypic methods of skin constructs have been used to mimic the *in vivo* environment. Butler et al. cultured a keratinocyte layer upon a fibroblast cell layer to mimic epidermal-dermal interface. They used this model to compare tissue thickness between keloid derived fibroblasts and normal fibroblasts, finding thicker growth of the artificial tissue in the keloid fibroblast group [69]. Artificial tissue constructs using rafts, consisting of fibroblasts embedded in a collagen gel with or without keratinocytes seeded on the surface, have been used in another organotypic culture model. After stratification of the keratinocyte layer, full-thickness incisions were made on the constructs, and these were maintained at air-fluid interface. Evaluation was performed with multiphoton microscopes that obtained serial images of multiple layers of the specimen and phase-contrast microscopy, enabling visualization of biologic activity of the wound [70]. Polylactic acid (PLA), polyglycolic acid (PGA), and their copolymers, polylactic-*co*-glycolic acid (PLGA), and polyhydroxybutyrate are biodegradable materials approved by the United States Food and Drug Administration for application in humans [71, 72]. The selection of the scaffold material, which composes the extracellular matrix of the tissue is a key to success of model establishment. Wang and Luo used PLGA because it is nontoxic, and the porosity rate is similar to human dermal structure [73]. 

Lee et al. applied the concept of multicellular tumor spheroid culture models to develop 3D organotypic multicellular keloid spheroids (OMSs) derived from freshly isolated keloid tissue and found that keloid characteristics and viability persisted for 7 days [74]. Bagabir et al. recently developed a method of long-term organ culture. Human keloid was dissected into 3,4,5, and 6 mm punch biopsy sizes, embedded in collagen gel, and then either submerged in serum-free and supplemented media (serum-free Dulbecco's modified Eagle's medium/Ham's F12 or William's E medium) or set in an air-liquid interface (ALI). They found that keloid tissue cultures in the ALI set in supplemented William's E (WE) medium most optimally expressed keloid characteristics up to 6 weeks [75].

### 2.3. Implantation of Tissue Engineered Scar Tissue into Athymic Mice

 Merging the techniques of tissue engineering and xenograft transplantation, recent studies have focused on the implantation of tissue engineered keloid tissue scaffolds or engineered skin substitutes into athymic mice. 

Yagi et al. developed an *ex vivo* glycosaminoglycan (GAG) deposition model, employing collagen sponges consisting of chemically cross-linked collagen resistant to collagenase digestion *in vivo*. These sponges were seeded with human keloid cells, then implanted in the subcutaneous space of nude mice. Sponges loaded with keloid lesion cells were compared with sponges seeded with fibroblasts harvested from normal skin. A month after implantation, the keloid sponge was significantly heavier than the fibroblast sponge, and this model was subsequently used to evaluate the effect of interleukin 1*β* or chondroitinase ABC, known to inhibit prostaglandins *in vitro* [22].

Wang and Luo, as previously mentioned, transferred human keloid fibroblasts to PLGA scaffolds sized 5 × 5 × 0.5 mm and cultured these in a rotator cell-culture system for one week. PLGA scaffolds without keloid fibroblasts were used as controls. These cultured scaffolds were implanted in a subcutaneous pouch on the backs of female athymic mice; the cell loaded scaffold on the left and the control in the right. The animals were sacrificed at day 30, 60, 120, and 180 for analysis. Keloid fibroblasts and collagen were observed at all time points, even at day 180 [73]. 

Supp et al. divided the dermis of a human keloid specimen into deep and superficial dermis, in order to assess the roles of deep and superficial keloid fibroblasts. They inoculated harvested and cultured keloid fibroblasts onto rehydrated bovine collagen-glycosaminoglycan polymer substrates, followed by keratinocytes. These engineered skin substitutes were incubated at the air-liquid interface for two weeks, then cut into 2 × 2 cm squares, and transplanted to full-thickness excision wounds cut on the right flank of nude athymic female mice. The grafts were sutured to the wounds and dressed with antimicrobial coated gauze, tied over with opposing sutures. The mice were photographed every 2 weeks and sacrificed at 12 weeks for analysis. The authors found that the group with deep fibroblasts had significantly thicker tissue, and that the group with superficial fibroblasts had significantly increased area [76].

Such methods provide a similar microenvironment for keloid tissue growth, and treatment modalities may be evaluated in a setting more closer to the *in vivo* environment. However, the keloid cells transferred to the scaffolds may still be obtained months to years after injury. The largest limitation is probably the requirement of both a sophisticated tissue engineering unit and qualified animal laboratory facilities.

## 3. Conclusion

This article describes the animal models utilized in abnormal scarring research to date. These models have provided valuable information about the pathogenesis and treatment possibilities of such scars. As with most other animal models, the validity of each of these models depends on the extent of similarity to human characteristics. However, because no model yet exactly replicates the pathophysiological condition *in vivo*, results analyzed from each study must be interpreted in the context of the model used. While recent progress merging tissue engineering with animal studies looks quite promising, there is still much to be done. Induction of keloids, not hypertrophic scars, on human skin grafted onto mice may be developed. Genetic models, which may enable us to finally analyze preventive measures, are likely to show up in the future. 
